# Personality traits and physical activity in middle-aged and older adults: a rapid review of evidence and interventions

**DOI:** 10.1093/geront/gnag173

**Published:** 2026-08-07

**Authors:** Keqin Chu, Jasmine Montayre, Patrick Pui Kin Kor, Jed Montayre

**Affiliations:** School of Nursing, The Hong Kong Polytechnic University, Hong Kong SAR, China; School of Nursing, The Hong Kong Polytechnic University, Hong Kong SAR, China; School of Nursing, The Hong Kong Polytechnic University, Hong Kong SAR, China; JBI Hong Kong Centre of Evidence-Based Healthcare Excellence, School of Nursing, The Hong Kong Polytechnic University, Hong Kong SAR, China; School of Nursing, The Hong Kong Polytechnic University, Hong Kong SAR, China; JBI Hong Kong Centre of Evidence-Based Healthcare Excellence, School of Nursing, The Hong Kong Polytechnic University, Hong Kong SAR, China

**Keywords:** Personality traits, Physical activity, Five-factor model, Health promotion

## Abstract

**Background and Objectives:**

Promoting physical activity (PA) is essential for health in middle-aged and older adults, yet global activity levels remain low. While personality is a recognized determinant of PA in the general adult population, personality-PA associations and personality-informed interventions in middle-aged and older adults remain under-explored. This rapid review aimed to synthesize evidence on Five-Factor Model personality traits and PA outcomes in adults aged ≥40 years and to explore trait-tailored interventions for this group.

**Research Design and Methods:**

Following PRISMA guidelines, systematic searches of PubMed, CINAHL, PsycINFO, and Cochrane Library were conducted through May 2025. A narrative synthesis approach was used to analyze eligible observational or intervention studies. Methodological quality was assessed using the Mixed Methods Appraisal Tool Version 2018.

**Results:**

19 studies met the inclusion criteria. Extraversion was consistently linked to higher PA across age groups, while neuroticism exhibited negative associations primarily in middle and older adulthood. Conscientiousness demonstrated positive relationships that strengthened with age, and openness was associated with PA primarily during transitional life stages. Evidence for agreeableness remained inconclusive. Only 2 PA interventions incorporated personality traits, highlighting a critical research gap. These preliminary findings suggest that intervention efficacy may depend on traits, with a digital self-control program benefiting highly conscientious individuals and an exergaming program particularly motivating less extraverted participants.

**Discussion and Implications:**

Personality traits influence PA engagement in middle-aged and older adults, with relationships varying by trait and life stage. These findings provide a foundation for developing personality-informed interventions aimed at enhancing PA engagement in aging populations.

The high prevalence of chronic conditions, such as heart disease, arthritis, depression, and diabetes, compromises well-being and quality of life in older adults (Griffith et al., 2017). These health challenges impose considerable societal burdens, including heightened healthcare demands and socioeconomic pressures (Rudnicka et al., 2020). Given the projection that the global population aged 65 years and above will surpass 1.5 billion by 2050 (United Nations, 2020), addressing these issues become increasingly urgent. Promoting physical activity in this population is critical, as it represents a well-established strategy for mitigating these health challenges.

## Physical activity in aging

Physical activity (PA), defined as bodily movement that requires energy expenditure (Caspersen et al., 1985), is fundamental for the development of effective health promotion strategies. It has demonstrated efficacy in managing chronic conditions, enhancing cardiovascular and musculoskeletal health, reducing fall risk, and improving psychological well-being (Chodzko-Zajko et al., 2009). Accordingly, the World Health Organization (WHO, 2020) recommends that older adults engage in either moderate-intensity exercise (150–300 min weekly) or vigorous-intensity exercise (75–150 min weekly), supplemented by muscle-strengthening exercises on two or more days. Despite these guidelines, PA participation declines as people age, with only around 10% to 30% of older adults worldwide meeting recommended levels (Bennie et al., 2017; Colley et al., 2011; Hansen et al., 2019). Middle-aged adults, though frequently understudied, represent a pivotal transitional phase, as health behaviors developed during this period may persist into later life when PA levels typically decline (Lachman et al., 2015; Mroczek et al., 2020). Identifying factors that influence PA in both middle and older adulthood is therefore essential for targeted health promotion strategies. These factors include health status, socioeconomic conditions, built environment, and psychological aspects, particularly personality traits.

## Personality traits as determinants of physical activity

The lifespan perspective on personality and health posits that personality influences long-term lifestyle patterns, including PA behavior (Friedman & Kern, 2014). Personality is conceptualized as a consistent constellation of traits that shape an individual’s thoughts, feelings, and actions (McAdams, 1995). It is considered a robust precursor of behavior, empowered by its predictive capabilities and practical relevance across various contexts (Laborde et al., 2020). The Capability, Opportunity, Motivation—Behavior (COM-B) model provides a useful framework for understanding how personality influences PA (Michie et al., 2011). This model posits that behavior arises from the interaction of capability, opportunity, and motivation (Michie et al., 2011). Within this framework, personality may differentially influence these components to shape PA behavior. For instance, traits such as neuroticism may undermine motivation through heightened negative affectivity (Olaru et al., 2023), while conscientiousness may enhance capability through self-regulatory skills (Rhodes et al., 2002).

Evidence from systematic reviews (e.g., Macali et al., 2025), meta-analyses (e.g., Wilson & Dishman, 2015), and empirical studies (e.g., Artese et al., 2017) has consistently demonstrated links between personality and PA, predominantly using the Five-Factor Model (FFM or “Big Five”; McCrae & Costa, 1992). The FFM has been extensively validated across ages, genders, and cultures, enhancing its applicability to diverse populations (Chapman et al., 2007; Komarraju et al., 2009). The model comprises five broad trait domains: conscientiousness (the inclination to be organized, disciplined, and goal-oriented), agreeableness (trust and cooperation), extraversion (sociability and outgoingness), openness (novelty-seeking and open-mindedness), and neuroticism (tendency toward negative emotions) (McCrae & Costa, 2008).

Among the FFM traits, conscientiousness, extraversion, and neuroticism exhibit the most robust associations with PA. Adults higher in conscientiousness and extraversion consistently show greater PA participation, whereas those with elevated neuroticism are less likely to be physically active (Macali et al., 2025; Rhodes & Smith, 2006; Sutin et al., 2016; Wilson & Dishman, 2015). These traits not only correlate with activity levels but also moderate the transition of behavioral intentions into actual PA and affect intervention effectiveness. Specifically, openness (Ma et al., 2022), conscientiousness, and extraversion (Rhodes & Dickau, 2013) have been identified as moderators of the link between intentions and PA behavior. Intervention studies further revealed that individuals higher in conscientiousness demonstrated greater improvements in daily step counts (e.g., Stieger et al., 2023). In contrast, findings on agreeableness and openness present more variability. A meta-analysis by Caille et al. (2024) reported positive associations of openness and agreeableness with PA initiation and adherence. However, Rhodes and Boudreau (2017) yielded mixed results, highlighting the need for further investigation of trait-activity relationships within the FFM framework.

## Gaps in current research

Despite extensive research on personality-PA links, several gaps remain. First, while conscientiousness, extraversion, and neuroticism demonstrate consistent associations with PA, findings for openness and agreeableness remain inconclusive (Caille et al., 2024; Rhodes & Boudreau, 2017). Second, existing reviews predominantly examine general adult populations, offering limited attention to developmental variations in middle and older adulthood. This gap is significant given age-related changes in personality traits, such as diminishing extraversion and increasing agreeableness (Debast et al., 2014), and barriers to PA in aging populations, such as declining functional health (Lachman et al., 2015). Third, current PA promotion strategies often adopt a generic “one-size-fits-all” approach (Chapman et al., 2014), which shows limited capacity to accommodate individual differences in requirements, preferences, and concerns (Kreuter et al., 1999). In contrast, tailored interventions that assess individual factors to select relevant components (e.g., exercise prescriptions) hold promise for enhancing PA engagement (Hill et al., 2015). Although emerging evidence supports the potential efficacy of personality-tailored interventions (Stieger et al., 2023), this approach remains understudied in middle-aged and older adults.

To address these gaps, a rapid review methodology was employed to provide a timely evidence synthesis (Saul et al., 2013). The FFM served as the guiding personality framework due to its extensive validation (Chapman et al., 2007; Komarraju et al., 2009) and prominence in health behavior research (Deary et al., 2010). The present review extends beyond existing syntheses in three key ways: first, by examining personality-PA relationships specifically in middle-aged and older adults; second, by identifying intervention studies that incorporate personality traits to promote PA; and third, by synthesizing evidence to help inform the future development of personality-tailored interventions.

## Aims and significance of the review

The primary aim of this review was to synthesize existing evidence on associations between the FFM personality traits and physical activity outcomes in middle-aged and older adults. The secondary aim was to explore physical activity interventions tailored to the FFM trait profiles in middle-aged and older adults.

By elucidating the relationships between specific personality trait domains and PA behavior, this review sought to provide insights for developing more precise, evidence-based interventions. Tailoring strategies to individual trait profiles may accommodate personality differences, enhance intervention efficacy, and ultimately support healthier aging trajectories.

## Method

This study employed a rapid review methodology, which streamlines the systematic review process by applying adaptations such as narrower database searching and a narrative synthesis (Tricco et al., 2015). This methodology was selected to provide a timely synthesis that informs intervention development (Ganann et al., 2010) and offers an overview of personality-PA associations and intervention characteristics, rather than seeking definitive evidence of intervention effectiveness. While these adaptations may result in less comprehensive coverage of the literature, research suggests that rapid reviews often yield conclusions comparable to full systematic reviews (Abou-Setta et al., 2017; Tricco et al., 2015), supporting their use as an efficient and structured synthesis tool.

The review adhered to the Preferred Reporting Items for Systematic Reviews and Meta-Analyses (PRISMA) guidelines (Moher et al., 2009) (see Table S1 for completed PRISMA checklist) and used the PEO (Population; Exposure; Outcome) framework, adapted to encompass both observational and interventional evidence. This review was pre-registered with the Open Science Framework (https://doi.org/10.17605/OSF.IO/HE4PR).

### Information sources

A comprehensive literature search was conducted across four electronic databases: PubMed (via the National Library of Medicine interface), CINAHL (via the EBSCO interface), APA PsycINFO (via the ProQuest interface), and Cochrane Library (see Table S2). Search terms were adapted for each database using specific keywords and index phrases.

### Search strategy

The development of the search strategy began with a preliminary PubMed search to identify relevant keywords. Following this, the strategy was refined by the review team and structured around three core domains: (a) physical activity concepts, (b) personality constructs, and (c) target population. The search terms were (physical activit* or exercise or fitness or movement or physical activity intervention* or exercise intervention* or physical activity program* or exercise program*) AND (personality or personality trait* or big five or five factor model or extraversion or conscientiousness or openness or agreeableness or neuroticism) AND (middle-aged and older adult* or middle and older adulthood or middle-aged adult* or middle aged or midlife or middle adulthood or older adult* or elderly or aging or ageing or older adulthood or later adulthood). These domains were combined using the Boolean operator “AND,” with terms within each domain connected by the operator “OR.” To capture the full breadth of available evidence, the search was applied to title and abstract fields and conducted in May 2025 without temporal restriction. Only English-language publications were considered due to resource constraints in translation.

### Eligibility criteria

#### Inclusion criteria

##### Population

Middle-aged and older adults aged 40 years and above in all settings.

##### Exposure

Adapted to encompass two aspects: (a) at least one of the Five-Factor Model personality traits (extraversion, conscientiousness, openness, agreeableness, and neuroticism), measured using a validated tool, and (b) receipt of trait-tailored PA intervention components.

##### Outcome

Measures of PA behavior (frequency, adherence, and intensity). Physical activity is defined as any bodily movement that requires energy expenditure (Caspersen et al., 1985). This includes, but is not limited to, exercise; sports; training; lifestyle activities (e.g., walking, gardening, and cycling for transport); and mind-body exercises (e.g., tai chi, yoga, and Pilates), where PA is a primary component. Outcomes should be assessed via self-report (e.g., questionnaire) or device-based means (e.g., accelerometers). Studies involving participants selected based on their engagement in the above forms of PA were also eligible.

##### Study design

Observational studies (e.g., cohort and cross-sectional studies) examining associations between personality traits and PA outcomes, and intervention studies (e.g., randomized controlled trials) evaluating trait-tailored PA interventions.

##### Language

English only.

##### Date

Searches were conducted from database inception to May 2025, with no date restriction applied.

#### Exclusion criteria

Studies were excluded if they focused exclusively on participants aged under 40 or if they included clinical populations with conditions that severely limit PA capacity (e.g., cognitive impairment, dementia). Studies that did not measure both PA and personality traits as primary variables were omitted. Animal model research, abstracts, protocols, commentaries, reviews, meta-analyses, gray literature (e.g., newsletters, dissertations, theses, conference presentations, or proceedings), and studies without full text were also excluded.

### Study selection

Prior to screening, the two reviewers coordinated to ensure they would apply the inclusion and exclusion criteria consistently. Records identified through initial database searches were imported into EndNote 21 (Clarivate Analytics, Philadelphia, PA) for preliminary management. Following the collection of full-text versions, all records were transferred to Covidence, a specialized systematic review management platform, for further processing. Duplicates were removed via automatic detection and manual verification. Two independent reviewers systematically evaluated the resulting records through a two-phase screening process. In the primary phase, reviewers screened titles and abstracts independently against the eligibility criteria. Full texts of potentially eligible articles were then independently assessed by the same reviewers during the secondary phase. The review team achieved agreement on all decisions, with a third reviewer present to address any potential discrepancy.

### Data extraction

Two independent reviewers extracted data using the data extraction form developed by the review team (see Table S3). The extraction tool captured the following key elements: general study characteristics (first author’s name, year of publication, title, country/area, context, aim(s)), characteristics of included studies (study design, study name, start time, end time), characteristics of participants (description, baseline age, total number, recruitment method, inclusion criteria, exclusion criteria), measures of variables (measure of exposure(s), measure of outcome(s)), intervention characteristics (components and delivery), and key findings. Any discrepancy between the reviewers was resolved through discussion or by involving an additional reviewer.

### Methodological quality appraisal

The methodological quality of included studies was assessed by one reviewer and subsequently verified by another using the Mixed Methods Appraisal Tool (MMAT) Version 2018 (Hong et al., 2018). The MMAT enables quality appraisal across diverse study designs within a review, accommodating qualitative, quantitative descriptive, quantitative non-randomized, quantitative randomized controlled, and mixed-methods studies. The tool comprises two initial screening questions followed by five design-specific quality criteria, each rated as “Yes,” “No,” or “Can’t tell.”

### Data synthesis and presentation

A narrative synthesis was conducted to map the available evidence, consistent with this review’s objective. Two reviewers independently performed the synthesis, summarizing descriptive data from all included studies in tables and figures. The original data extraction form was simplified for the *Results* section, which contained elements including author, year, area, setting, study design, participant characteristics, PA measures, personality measures, and key findings. Study findings were categorized by personality trait and age cohort, with the direction of association quantified through proportional analysis. Four age groups were established: young to older transition (≥19 years), middle-aged adults (40–65 years), middle-aged to older transition (≥40 years), and older adults (≥65 years). Erikson’s theory of psychosocial development, young adulthood (19–40 years), middle adulthood (40–65 years), and late adulthood (≥65 years) (Erikson, 1994), provides the theoretical foundation for the age categorization. The two transitional categories (young-to-older and middle-aged-to-older) were formed to accommodate studies with samples spanning multiple life stages. Within each age category, the direction of association (positive, negative, or null) between personality traits and PA was summarized. This was recorded as the proportion of studies reporting a specific direction (“X out of Y studies”), where Y represents the total number of studies for that trait within the age group. The synthesized patterns are visually represented in Figure 4 and further elucidated in a narrative summary.

## Results

**Figure 1:**
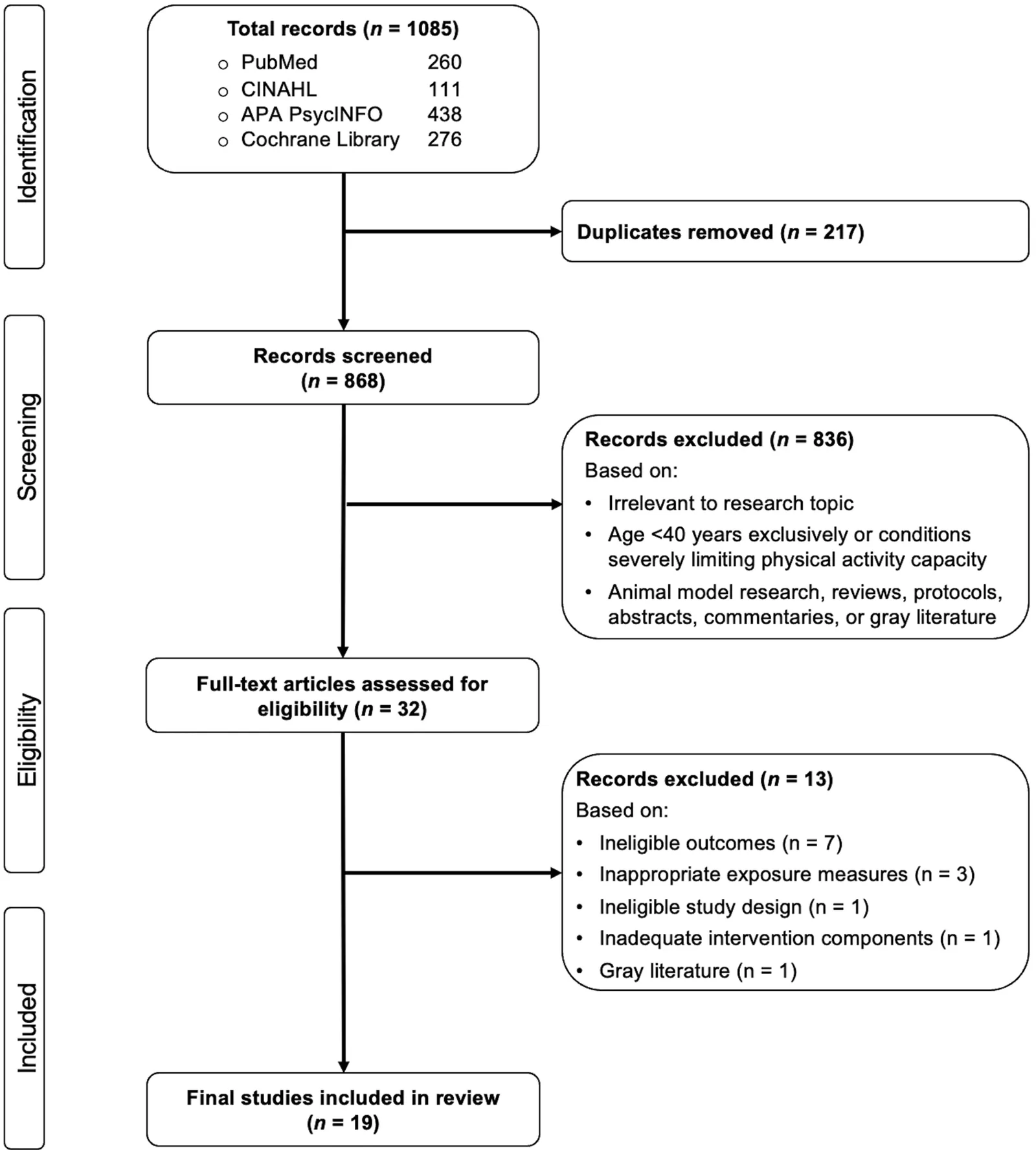
PRISMA flowchart for selection of studies.

The initial literature search identified 1,085 records. Following the removal of duplicates (*n* = 217), 868 records underwent title and abstract screening, which identified 32 articles for full-text review. After excluding studies with ineligible outcomes (*n* = 7), inappropriate measures for exposures (*n* = 3), inadequate intervention components (*n* = 1), ineligible study design (*n* = 1), or classification as gray literature (*n* = 1), a total of 19 studies met the inclusion criteria (see Figure 1).

### Characteristics of included studies

**Figure 2:**
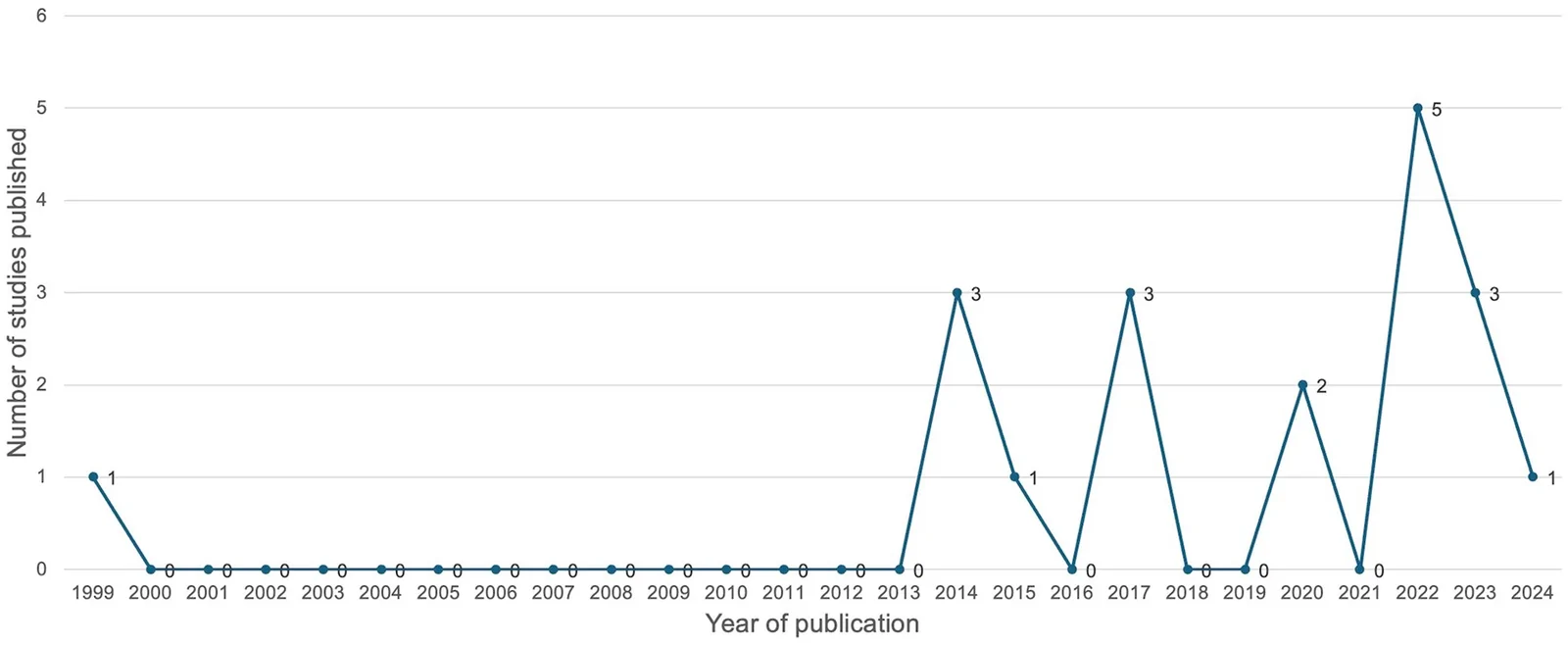
Number of studies included in the review by year of publication (1999–2024).

**Figure 3:**
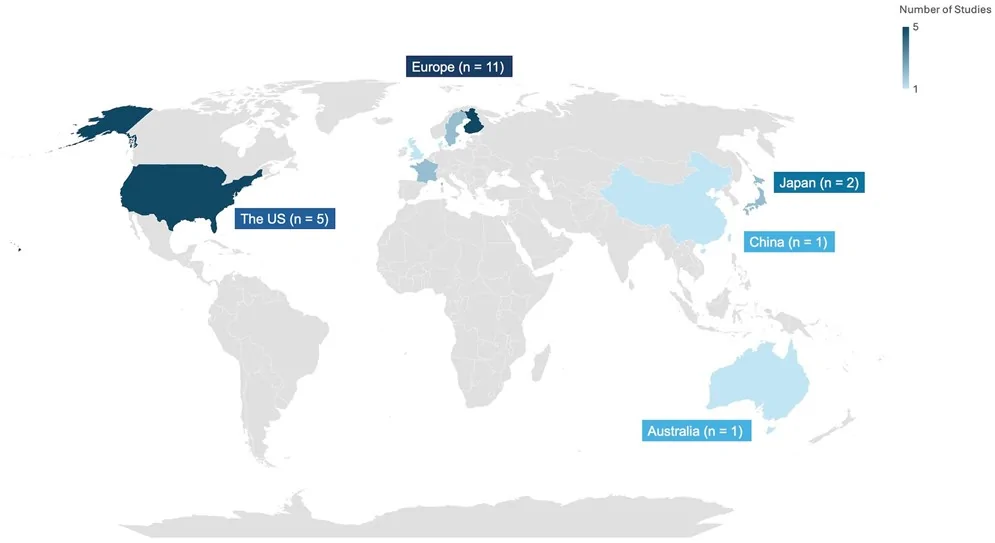
Number of studies included in the review by country.

Included studies were published between 1999 and 2024, with a noticeable increasing trend in research exploring personality and PA over this period (see Figure 2). Geographically, most studies were conducted in Europe (*n* = 10), with Finland contributing the highest number (*n* = 4), followed by Sweden (*n* = 2), and single studies from Denmark, France, and the United Kingdom, as well as one study covering multiple European countries. North America was represented by four studies, all from the United States. Three studies were carried out in Asia, specifically in Japan (*n* = 2) and China (*n* = 1). Additionally, one study was based in Australia (Oceania), and another used multinational data from France to the United States (see Figure 3). All studies were community-based, with cross-sectional study designs being the most common (*n* = 11). The remaining studies included longitudinal (*n* = 6) and intervention (*n* = 2) designs. Sample sizes varied widely, ranging from 16 to 28,304 participants. Female participants were predominant across most studies, with fourteen of the nineteen studies including more than 50% women. Among the fourteen studies that reported education levels, twelve found that the largest proportion of participants had taken upper secondary, high school, or vocational education. Socioeconomic data (income or occupation) were reported in only five studies, with participants spanning a broad range of status. Further details are listed in Table 1.

**Table 1:** Summary of included studies.

No.	Author (year) area	Setting Study design	Participant characteristics	Physical activity measures	Personality measures	Key findings
1	Artese et al. (2017) United States	Community Cross-sectional cohort study	Older adults 67–95 years; *N* = 69; female: 52 (75%); male: 17 (25%); socioeconomic factors not reported	The ActiGraph ActiSleep monitor	NEO-PI-3FH	Older adults who scored *higher in conscientiousness and extraversion* and *lower in neuroticism* have *greater levels of PA*. *High agreeableness* was associated with *more PA*.
2	Bhattacharyya et al. (2022) United States	Community Longitudinal cohort study	Young to older adults 33–83 years; age *M* = 55 (*SD* = 11); *N* = 2,050; female: 56%; male: 44%; 96% ≥ high school; 58% employed	Mind-Body practice self-report	25 self-descriptive adjective items from the MIDI	The findings did not show any association between personality traits and subsequent MBP.
3	Emile et al. (2014) France	Community Cross-sectional survey	Middle-aged to older adults 60–93 years; *N* = 192; female: 114 (59%); male: 78 (41%); 31.8% < Baccalaureate; 40% Baccalaureate; 29% university degree	Dijon physical activity score	10-item subscale of the BFI-Fr	*Openness* to experience *positively predicted physical activity level* through incremental theories, endorsement of aging stereotypes relative to benefits, attitude toward own aging, and physical self-worth.
4	Kekäläinen, Laakkonen, et al. (2020) Finland	Community Cross-sectional cohort study	Middle-aged and older adults (1) 47–55 years; *N* = 1,098; female: 100%; 41% university degree (2) 70–85 years; *N* = 314; female: 188 (60%); male: 126 (40%); 21% university degree	Physical activity self-report and tri-axial accelerometers	19-item short form of the Eysenck personality inventory	Among middle-aged women, those *higher in extraversion and lower in neuroticism* reported *higher levels of PA*. Among these women, *lower scores in neuroticism* also had a *weak negative* association with *accelerometer-measured leisure time moderate-to-vigorous PA*. No associations of extraversion or neuroticism with physical activity were found in the older men and women.
5	Kekäläinen et al. (2020) Finland	Community Cross-sectional cohort study	Older adults 70–85 years; *N* = 239; female: 141 (59%); male: 98 (41%); 12% low (≤ primary); 66% medium (middle/vocational/secondary); 22% high (college/university) education	Physical activity self-report and triaxial accelerometer	240-item NEO personality inventory-3	Extraversion was *positively* associated with *self-reported metabolic equivalent minutes (MET-minutes)*, and conscientiousness with *both light physical activity and self-reported MET-minutes*.
6	Kekäläinen et al. (2022a) European countries	Community Cross-sectional cohort study	Middle-aged to older adults ≥50 years; self-reported PA: *N* = 46,617; device-based PA: *N* = 855; female: ∼57%; male: ∼43% (across countries); average education ≈3.0 (upper secondary)	Physical activity self-report and triaxial accelerometer	10-item BFI	Only *conscientiousness* was *positively* associated with *intensity distribution*. Adults with *less resilient* personality traits living in countries with lower resources are at the *highest risk for physical inactivity.*
7	Kekäläinen et al. (2022b) Finland	Community Longitudinal cohort study	Middle-aged adults 47–55 years; *N* = 441; female: 100%; 1% lower secondary; 55% upper secondary; 44% tertiary or higher education	Triaxial GT3X and wGT3X ActiGraph accelerometers	Modified short form of the Eysenck personality inventory	*Neuroticism* was negatively associated with autonomous motivation and had a *negative indirect* association with *moderate-to-vigorous leisure-time physical activity (MVPA)* through autonomous motivation.
8	Kekäläinen et al. (2023) Finland	Community Longitudinal cohort study	Young to older adults Followed from 8 to 42 and 50 years; *N* = 369; female: 173 (47%); male: 196 (53%); average education at age 27: vocational school	Physical activity self-report	NEO personality inventory	Adulthood *extraversion* predicted midlife *physical activity* in women. Neither conscientiousness nor neuroticism in adulthood was associated with physical activity.
9	Marks and Lutgendorf (1999) United States	Community Cross-sectional study	Older adults ≥65 years; *N* = 97; female: 68 (70%); male: 29 (30%); 21% < high school; 30% high school; 49% post-high school; income: mean reported $10,000-$20,000 annually	24-item PLQ	54-item BFI	Individuals with *greater conscientiousness* demonstrated significantly greater PHC, *higher levels of exercise*, and more relaxation/social support behaviors.
10	Olaru et al. (2023) Sweden	Community Longitudinal cohort study	Middle-aged to older adults ≥60 years; *N* = 2,694; female: 1,670 (67%); male: 824 (33%); average education >10 years	Physical activity self-report	36 items out of the 60 items on the NEO-FFI	In both groups, *lower neuroticism, higher extraversion, and higher openness levels* were moderately associated with *stronger engagement in all types of activities (including physical activity)*.
11	Petersen et al. (2018) Denmark	Community Cross-sectional cohort study	Middle-aged adults 48–62 years; *N* = 4,649; female: 1,410 (30%); male: 3,239 (70%); occupational social class: 47% high; 53% low	IPAQ-LF	Danish short version of the NEO-FFI	*Extraversion* was the personality trait most consistently and *positively* associated with *higher duration of physical activity*, while *conscientiousness, extraversion, neuroticism, and openness* were *positively* associated with *intensity*.
12	Sjöberg et al. (2022) Sweden	Community Longitudinal cohort study	Older adults 65–99 years; *N* = 624; female: 413 (66%); male: 211 (34%); 42% ≤ high school; 58% > high school	Physical activity self-report	36-item short version of the NEO-FFI	The pre-pandemic presence of *higher levels of neuroticism* was associated with *reductions in higher-intensity PA* during the pandemic.
13	Stephan et al. (2014) United States and France	Community Cross-sectional cohort study	Young to older adults (1) 30–84 years; age *M* = 55 (*SD* = 12); *N* = 3,396; female: 54%; male: 46%; 68% > high school (2) 30–84 years; mean age = 59 years (*SD* = 13 years); *N* = 2,917; female: 64%; male: 36%; 73% > high school	Physical activity self-report	(1) MIDI (2) BFI-Fr	In both samples, individuals who scored *higher on extraversion and openness* were more likely to engage in *a variety of physical*, cognitive, and social *activities*.
14	Steptoe et al. (2017) United Kingdom	Community Cross-sectional cohort study	Middle-aged to older adults ≥50 years; *N* = 2,318; female = 1,374 (59%); male = 944 (41%); 20% no qualifications; 42% basic/high school; 38% college/graduate; wealth: equal distribution from lowest to highest	Physical activity self-report	MIDI personality scales	Consciousness was associated with healthier lifestyle choices, with *more conscientious* individuals being *physically active non-sedentary*.
15	Stieger et al. (2023) United States	Community Two-arm randomized controlled trial	Middle-aged adults 35–65 years; *N* = 86; female: 78%; male: 22%; average education: 17 years	IPAQ-SF and Fitbit charge 4 activity trackers	60-item BFI-2	Participants with *higher initial levels of conscientiousness* were better able to *increase their daily steps* during the intervention. Those of the treatment group with *higher initial levels in conscientiousness* or greater increases in self-control were better able to *increase their physical activity* as compared to those of the comparison group.
16	Thøgersen-Ntoumani et al. (2022) Australia	Community Longitudinal study	Middle-aged adults 40–65 years; *N* = 297; female: 72%; male: 28%; 78% are in a relationship	IPAQ-SF	20-item API	Conscientiousness did not predict PA at T2. *Neuroticism* predicted *low levels of PA*.
17	Wang et al. (2024) China	Community Cross-sectional survey	Young to older adults 20–96 years; middle adulthood (age *M* = 50, *SD* = 8); older adulthood (age *M* = 71, *SD* = 5); *N* = 28,304; female: 50%; male: 50%; 87% without associate degree; 74% employed; income: 24% ¥10,000–49,999	Physical activity self-report	15‐item version of the BFI	There were more exercisers among resilient individuals across adulthood and among *confident* individuals in later adulthood; *more non‐exercisers* appeared among *vulnerable* individuals. As for exercise duration, the *resilient* profiles showed *longer exercise duration* in young and middle adulthood; the *confident* profiles showed *longer exercise duration* in middle and later adulthood.
18	Yasunaga and Yaguchi (2014) Japan	Community Cross-sectional survey	Middle-aged to older adults 60–92 years; *N* = 876; female: 389 (44%); male: 487 (56%); socioeconomic factors not reported	The exercise/sports domain of PAQ-EJ	Japanese version of the NEO-FFI	*Extraversion and conscientiousness* were significantly and *positively* associated with *exercise level* after controlling for age, sex, and physical health. However, neuroticism, openness, and agreeableness were not significantly associated with exercise level.
19	Zaitsu et al. (2015) Japan	Community Within-subject intervention study	Older adults 65–82 years; *N* = 16; male: 100%; socioeconomic factors not reported	LTEQ 15	Japanese version of the 60-item Big Five Scale	The difference between the *average number of times that exercise* occurred with and without exergame use was *positively* correlated with *neuroticism*, *negatively* correlated with *extraversion*, and not associated with conscientiousness. *Less extraverted* individuals were more likely to *perform the sit-to-stand exercise* when using the exergame, whereas *more extraverted* individuals *performed the exercise* regardless of the intervention conditions.

*Note*. Age *M* = mean age in years; API = Australian Personality Inventory; BFI = Big Five Inventory; BFI-Fr = the French version of the Big Five Inventory; IPAQ = International Physical Activity Questionnaire; IPAQ-LF = International Physical Activity Questionnaire Long Form; IPAQ-SF = International Physical Activity Questionnaire Short Form; LTEQ = Leisure Time Exercise Questionnaire; MBP = mind-body practice; MIDI = Midlife Development Inventory; NEO-FFI = NEO Five-Factor Inventory; NEO-PI-3FH = NEO Personality Inventory-3 First Half; PA = physical activity; PAQ-EJ = Physical Activity Questionnaire for Elderly Japanese; PHC = perceived health competence; PLQ = Personal Lifestyle Questionnaire.

### Associations between personality traits and physical activity

The most frequently used measures of PA in the included studies were the International Physical Activity Questionnaire (IPAQ) and tri-axial accelerometers, each used in four studies. Types of PA included yoga, Pilates, tai chi, Qigong, aerobics, ball sports, water sports, ice sports, snow sports, contact sports, skating, cycling, running, gymnastics, dancing, weight training, housework including laundry, home repairs, gardening, and cleaning, jogging, mountaineering, walking, and going hunting or fishing. For assessing personality traits, the predominant tools were the Big Five Inventory (BFI; John & Srivastava, 1999), a 44-item measure of five broad traits, and the NEO Personality Inventory (NEO-PI; McCrae & Costa, 1992), a 240-item instrument measuring five broad traits and 30 subfacets. Each tool was used in seven studies. The five broad traits included neuroticism, extraversion, conscientiousness, agreeableness, and openness. As shown in Table 1, all five personality traits demonstrated associations with PA, though the consistency of these relationships varied. Specifically, higher levels of extraversion and conscientiousness were consistently linked to increased PA. Conversely, neuroticism exhibited negative associations with PA engagement. Openness showed less consistent, positive relationships, and evidence for agreeableness remained limited.

**Figure 4:**
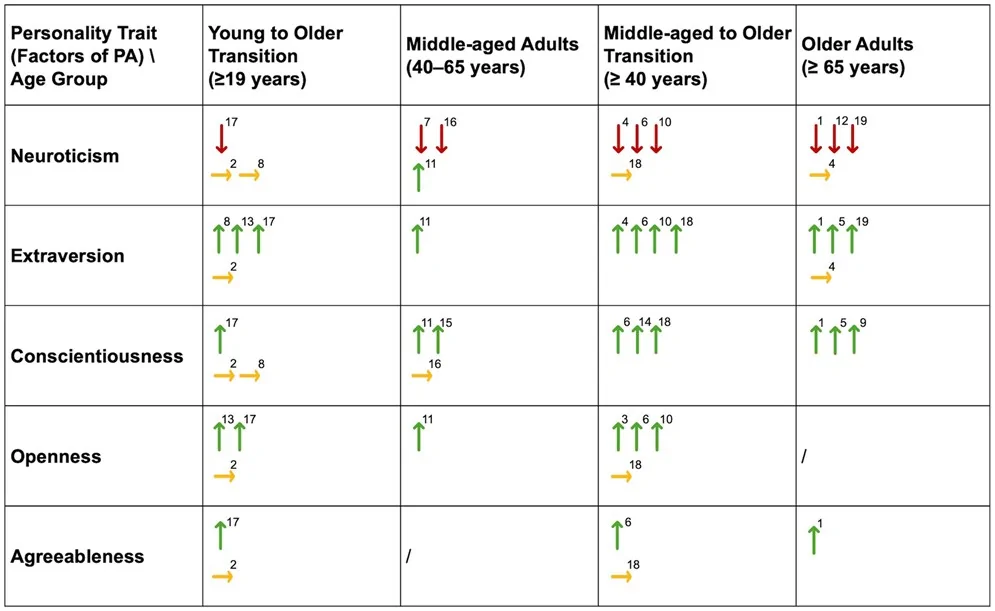
Associations between FFM personality traits and physical activity across age groups. *Note*. Arrows indicate the direction of the association between personality traits and physical activity. Upward arrows denote a positive association, downward arrows denote a negative association, and horizontal arrows indicate a null association. The number of arrows represents the number of studies reporting the specific result. Superscripts correspond to specific studies numbered in Table 1. FFM = Five-Factor Model; PA = physical activity.

After categorizing participants into different age groups, the analysis revealed age-specific patterns in associations between personality traits and PA (see Figure 4). Neuroticism showed no effect during the young-to-older transition period (2/3 studies show →) but demonstrated negative associations with PA in middle-aged adults (2/3 studies show ↓), middle-aged-to-older transition (3/4 studies ↓), and older adults (3/4 studies ↓). Nevertheless, a positive relationship between neuroticism and PA intensity was found in middle-aged adults. These results indicated its role as a barrier to PA in later life stages. Extraversion showed the most robust evidence base for PA promotion, with multiple studies reporting positive associations at every life stage (Young-Older Transition: 3/4 studies ↑; Middle-aged Adults: 1/1 study ↑; Middle-Older Transition: 4/4 studies ↑; Older Adults: 3/4 studies ↑). For conscientiousness, the consistency of positive associations strengthened during later life transitions (Middle-Older Transition: 3/3 studies ↑ vs Young-Older Transition: 1/3 studies ↑), highlighting its increasing importance with age. Openness showed positive associations during transitional life phases (Young-Older: 2/3 studies ↑; Middle-Older: 3/4 studies ↑) and middle adulthood (1/1 study ↑), whereas no finding was reported in older adulthood. Agreeableness presented limited but positive evidence (3/5 total studies ↑), except in transitional life stages where no association was observed (2/5 total studies).

### Intervention studies

Two intervention studies examined how personality traits influenced responses to PA interventions (Stieger et al., 2023; Zaitsu et al., 2015). Stieger et al. (2023) evaluated a 7-week smartphone-based self-control intervention among middle-aged adults, employing chatbot-based coaching and behavioral tasks grounded in the process model of self-control. The intervention group demonstrated greater increases in self-reported PA compared to the control group, with participants higher in baseline conscientiousness showing greater improvements in daily step counts. Zaitsu et al. (2015) examined a 12-week exergame intervention using a movement tracking device in older adults. While no significant difference in exercise performance was observed between the exergame and self-regulated conditions, individuals with lower extraversion were more likely to perform exercise during the exergame condition.

### Methodological quality appraisal

The results of the methodological quality appraisal are provided in Table S4. While all 19 included studies fulfilled the two initial screening criteria, adherence to the five design-specific quality criteria varied. Specifically, only one study met all five criteria. Most of the remaining studies met either four (*N* = 7) or three (*N* = 8) criteria, with the final three studies fulfilling only two criteria.

## Discussion

This rapid review synthesized evidence on the associations between personality traits and physical activity in middle-aged and older adults, revealing distinct age-specific patterns. Extraversion demonstrated a consistent positive association across age groups, while neuroticism showed a negative association primarily in middle and late adulthood. For conscientiousness, the positive relationship became increasingly robust with age, with the most consistent evidence emerging during the transition into older adulthood. Phase-contingent relationships were noted for openness, which linked positively with PA primarily during transitional life stages (e.g., middle-to-older adulthood). In contrast, evidence for agreeableness remained inconclusive. Furthermore, while personality-informed interventions remain limited, preliminary evidence from community settings suggests that intervention efficacy may depend on individual traits. Collectively, these findings underscore the role of personality in PA engagement and highlight the need for future research to investigate how trait profiles might inform tailored PA promotion strategies for middle-aged and older adults.

### Neuroticism

The present review found a consistent negative association between neuroticism and PA engagement in middle-aged and older adults, aligning with previous evidence (e.g., Rhodes & Smith, 2006; Sutin et al., 2016; Wilson & Dishman, 2015). Individuals high in neuroticism tend to demonstrate weaker exercise regulation motives (Costa et al., 2014) and face greater PA barriers, including motivational deficits, embarrassment during exercise, and chronic fatigue (Courneya & Hellsten, 1998). These behavioral and psychological barriers may originate from neuroticism’s fundamental characteristics of heightened negative affectivity and emotional instability (Olaru et al., 2023). Such traits may contribute to reduced PA engagement, particularly if the exercise-induced physiological arousal is interpreted as aversive or threatening (Wilson & Dishman, 2015). An exception was noted in midlife, where neuroticism showed a positive association with PA intensity (Petersen et al., 2018). This finding may reflect a compensatory coping mechanism, whereby midlife stressors (e.g., occupational demands, familial responsibilities) may drive individuals high in neuroticism to engage in intensive PA as a maladaptive regulation strategy.

### Extraversion

Extraversion emerged as the most robust correlate of PA across age groups, corroborating extensive research (e.g., Macali et al., 2025; Sutin et al., 2016) and underscoring its role in motivating sustained PA behavior. The positive link between extraversion and PA is likely driven by key characteristics of this trait, including high energy, sociability, and excitement-seeking (Stephan et al., 2014). People with these inherent tendencies are more inclined to engage in activities that provide sensory stimulation, social interaction, and physical exertion, all of which are common features of exercise (Wilson & Dishman, 2015). This inherent motivation may also explain why extraverts are less dependent on specific intervention formats. As one exergame intervention study demonstrated, more extraverted individuals performed exercise consistently across the intervention conditions, whereas their less extraverted counterparts showed better performance in the exergame condition than in the self-regulated condition (Zaitsu et al., 2015). These findings reinforce that extraverts’ PA engagement is primarily driven by self-determined motivational regulation (Costa et al., 2014), fulfilling their dispositional needs for energetic movement and social engagement. This, in turn, promotes their mood and reinforces sustained exercise behavior (Berger & Motl, 2000).

Despite consistent findings, two studies reported null associations between extraversion and PA engagement, including one study spanning young to older adults (Bhattacharyya et al., 2022) and another in older adults specifically (Kekäläinen et al., 2020). The discrepancy might be attributable to the type of PA measured. For instance, the study of Bhattacharyya et al. (2022) assessed mind-body practices (e.g., yoga, Pilates, and tai chi), which emphasize internal focus and calm, whereas extraversion is more robustly linked to high-energy, high-risk activities that align with the trait’s excitement-seeking nature (McEwan et al., 2019).

### Conscientiousness

Similar to extraversion, we found a positive relationship between conscientiousness and PA engagement, supported by previous findings (e.g., Rhodes & Smith, 2006; Wilson & Dishman, 2015). Individuals high in conscientiousness show characteristic discipline, deliberation, and a strong sense of duty (Bogg & Roberts, 2004). These traits facilitate greater engagement in a variety of health behaviors, including PA, through their perceived utility and social desirability (Bogg & Roberts, 2013). Furthermore, the enhanced capability of self-regulation associated with conscientiousness contributes to both the initiation and maintenance of PA behavior (Rhodes et al., 2002). Notably, this mechanism is corroborated by intervention research, which found that participants with higher baseline conscientiousness were more successful at increasing their daily steps, and those with improved self-control showed greater gains in PA intensity, highlighting the trait’s potential role in behavior change (Stieger et al., 2023).

While conscientiousness consistently facilitates PA, this review extends prior work by demonstrating an age-amplified effect, with more consistent associations revealed during later-life transitions. This developmental pattern aligns with evidence of increasing conscientiousness in middle-aged and older adults (Robinson et al., 2021). According to Roberts and his colleagues’ assumptions, this growth could be attributed to two key processes (Roberts et al., 2005). First, as individuals age, they gradually recognize and internalize their behavior change. Second, they adapt to the demands of major social roles, which occur through commitment to normative social institutions, such as employment and marriage. As these roles integrate into one’s identity, they introduce responsibility norms that reinforce conscientious behaviors.

### Openness

Openness showed positive but less consistent associations with PA compared to extraversion, conscientiousness, or neuroticism. While pre-existing evidence remains mixed, our findings are in line with previous reviews reporting this relationship (e.g., Sutin et al., 2016; Wilson & Dishman, 2015). It is suggested that individuals high in openness tend to demonstrate receptivity to novel ideas and opportunities, typically exhibiting greater willingness to explore diverse types of PA (Wilson & Dishman, 2015). Notably, current results showed that positive relationships were most evident during transitional life phases: young-to-older and middle-to-older adulthood. This developmental pattern corresponds with findings that openness promotes health behavior adaptation during critical aging transitions (Turiano et al., 2012) through greater willingness to adopt new routines. However, research on the openness-PA relationship in older populations remains limited (Stephan, 2009). This gap underscores the need for gerontological research on openness, as older adults high in openness are more inclined to participate in health-preserving and function-maintaining activities (Duberstein et al., 2003).

### Agreeableness

The association between agreeableness and PA engagement remains inconsistent. While three studies reported positive associations (Artese et al., 2017; Kekäläinen et al., 2022a; Wang et al., 2024), others found no association (Bhattacharyya et al., 2022; Yasunaga & Yaguchi, 2014). The proposed reason for positive findings is that older adults with high agreeableness, characterized by kindness and compassion, may accumulate more PA through assisting less mobile peers in aging populations, where functional limitations are common (Artese et al., 2017). This inconsistency extends to previous literature. For example, a meta-analysis by Rhodes and Smith (2006) found no overall relationship, whereas Caille et al. (2024) reported longitudinal associations of higher agreeableness with PA initiation and maintenance. Such divergent findings suggest that agreeableness may be less consistently associated with PA than other FFM traits, highlighting the need for further research to explore the potential moderating factors.

### Strengths and limitations

The present review has several strengths. It efficiently synthesized evidence from both observational and intervention studies. Structured approaches were employed, with screening and data extraction performed independently by two reviewers and quality appraisal by one reviewer, subsequently verified by another. The consistent associations observed across diverse methodological approaches, varied personality assessments, PA measures, and follow-up durations reinforce the robustness of these findings. Furthermore, this research extends current knowledge by demonstrating age-specific patterns in personality-PA relationships, highlighting how trait effects vary across middle and older adulthood. Notably, a paucity of personality-tailored interventions was identified, highlighting a critical research gap. Nevertheless, the limited but exploratory findings from the two identified interventions provide preliminary indications that aligning intervention content with trait profiles may represent a viable approach. Such tailoring could extend beyond generic “one-size-fits-all” strategies.

However, these findings should be interpreted in the context of the study’s limitations. First, the rapid review approach streamlined some methodological elements, including fewer search databases, an English-language restriction, and a narrative synthesis, which may have resulted in less comprehensive evidence coverage and potential interpretation bias. Second, methodological heterogeneity across studies, such as variations in PA assessment, study design, follow-up length, and sample size, may affect the comparability of results. Third, while the review focused on broad trait domains, facet-level analyses could bring insights into specific trait components most strongly associated with PA. Additionally, participants in some studies were classified as “physically active” if they reported any PA (e.g., Wang et al., 2024), potentially including individuals below WHO-recommended activity levels (World Health Organization, 2020). Future work should define a threshold using standardized metrics (PA frequency, duration, and intensity) that align with the WHO guidelines. Fourth, the predominance of observational studies precludes causal inferences of reported associations. Moreover, longitudinal evidence indicates that PA engagement could reciprocally influence personality development (Stephan et al., 2018), warranting future investigation of potential bidirectional relationships. Finally, this review is limited by geographic diversity and an overrepresentation of female and moderately educated participants. To enhance generalizability, future research should target underrepresented groups, specifically men and people with lower educational attainment, while expanding to regions such as Asia, Oceania, and South America.

### Implications for future research

The findings of this rapid review may have important implications for healthcare and future research. The robust negative associations between neuroticism and PA suggest that personality assessment could help identify individuals at heightened risk of inactivity, paving the way for targeted prevention using personality-appropriate techniques (Belmon et al., 2015). For example, individuals high in neuroticism may benefit from strategies that reduce exercise-related anxiety and improve self-regulation, such as mindfulness-based approaches (Thøgersen-Ntoumani et al., 2022). Similarly, those low in extraversion may respond better to gamified interventions that enhance engagement (Zaitsu et al., 2015). This trait-based approach provides a preliminary basis for identifying strategies that may promote adherence, moving beyond self-reported preferences alone. Future research would benefit from mixed-methods approaches to identify techniques tailored to specific trait profiles, thereby informing the development of evidence-based, targeted PA interventions.

Despite the potential of this tailored approach, its feasibility and cost-effectiveness warrant careful consideration. Implementation in community or healthcare settings presents logistical challenges, including the time burden of personality screening and the training required for interventionists to deliver personality-tailored techniques. Furthermore, cost-effectiveness remains uncertain, as the potential for improved health outcomes should be weighed against increased resources required for implementation. Consequently, future research should prioritize pilot studies to evaluate the feasibility, acceptability, and cost-effectiveness of personality-informed PA interventions before progressing to full-scale trials.

## Conclusions

This rapid review highlights the role of personality traits in PA engagement in middle-aged and older adults. The findings demonstrate that higher levels of extraversion, conscientiousness, and openness act as protective factors for PA, whereas neuroticism emerges as a barrier. The relationship between agreeableness and PA remains inconclusive, warranting further investigations. Notably, a substantial gap exists in the development and testing of personality-tailored PA interventions. Future research exploring targeted programs that account for individual trait profiles represents a potential avenue for enhancing intervention efficacy and promoting PA in aging populations.

## Supplementary Material

gnag173_Supplementary_Data

## Data Availability

The literature included in this review can be accessed via the cited references. The full search strategy and data extraction table are available in the Supplementary Material. Our analytical methods are available to other researchers and can be accessed upon request. This review was pre-registered with the Open Science Framework (https://doi.org/10.17605/OSF.IO/HE4PR).

## References

[gnag173-B1] Abou-Setta A. M. , Jeyaraman M., Attia A., Al-Inany H. G., Ferri M., Ansari M. T., Garritty C. M., Bond K., Norris S. L. (2017). Methods for developing evidence reviews in short periods of time: A scoping review. PLOS One, 12, e0165903. 10.1371/journal.pone.0165903 PMC5145149 27930662

[gnag173-B2] Artese A. , Ehley D., Sutin A. R., Terracciano A. (2017). Personality and actigraphy-measured physical activity in older adults. Psychology and Aging, 32, 131–138. 10.1037/pag0000158 28287783 PMC5369413

[gnag173-B3] Belmon L. S. , Middelweerd A., Te Velde S. J., Brug J. (2015). Dutch young adults’ ratings of behavior change techniques applied in mobile phone apps to promote physical activity: A cross-sectional survey. JMIR mHealth and uHealth, 3, e4383. 10.2196/mhealth.4383 PMC4704888 26563744

[gnag173-B4] Bennie J. , Pedisic Z., Suni J., Tokola K., Husu P., Biddle S., Vasankari T. (2017). Self‐reported health‐enhancing physical activity recommendation adherence among 64,380 Finnish adults. Scandinavian Journal of Medicine & Science in Sports, 27, 1842–1853. 10.1111/sms.12863 28230924

[gnag173-B5] Berger B. G. , Motl R. W. (2000). Exercise and mood: A selective review and synthesis of research employing the profile of mood states. Journal of Applied Sport Psychology, 12, 69–92. 10.1080/10413200008404214

[gnag173-B6] Bhattacharyya K. K. , Dobbs D., Hueluer G. (2022). Mind-body practice, personality traits, and cognitive performance: A 10-year study in US adults. Gerontology & Geriatric Medicine, 8, 23337214221083475. 10.1177/23337214221083475 35299879 PMC8922208

[gnag173-B7] Bogg T. , Roberts B. W. (2004). Conscientiousness and health-related behaviors: A meta-analysis of the leading behavioral contributors to mortality. Psychological Bulletin, 130, 887–919. 10.1037/0033-2909.130.6.887 15535742

[gnag173-B8] Bogg T. , Roberts B. W. (2013). The case for conscientiousness: Evidence and implications for a personality trait marker of health and longevity. Annals of Behavioral Medicine: a Publication of the Society of Behavioral Medicine, 45, 278–288. 10.1007/s12160-012-9454-6 23225322 PMC3604184

[gnag173-B9] Caille P. , Stephan Y., Sutin A. R., Luchetti M., Canada B., Heraud N., Terracciano A. (2024). Personality and change in physical activity across 3–10 years. Psychology & Health, 39, 670–690. 10.1080/08870446.2022.2092866 35765986 PMC9841291

[gnag173-B10] Caspersen C. J. , Powell K. E., Christenson G. M. (1985). Physical activity, exercise, and physical fitness: Definitions and distinctions for health-related research. Public Health Reports (Washington, D.C.: 1974), 100, 126–131. https://pmc.ncbi.nlm.nih.gov/articles/PMC1424733/3920711 PMC1424733

[gnag173-B11] Chapman B. P. , Duberstein P. R., Sörensen S., Lyness J. M. (2007). Gender differences in five-factor model personality traits in an elderly cohort. Personality and Individual Differences, 43, 1594–1603. 10.1016/j.paid.2007.04.028 18836509 PMC2031866

[gnag173-B12] Chapman B. P. , Hampson S., Clarkin J. (2014). Personality-informed interventions for healthy aging: Conclusions from a National Institute on Aging work group. Developmental Psychology, 50, 1426–1441. 10.1037/a00335 23978300 PMC3940665

[gnag173-B13] Chodzko-Zajko W. J. , Proctor D. N., Singh M. A. F., Minson C. T., Nigg C. R., Salem G. J., Skinner J. S. (2009). Exercise and physical activity for older adults. Medicine & Science in Sports & Exercise, 41, 1510–1530. 10.1249/MSS.0b013e3181a0c95c 19516148

[gnag173-B14] Colley R. C. , Garriguet D., Janssen I., Craig C. L., Clarke J., Tremblay M. S. (2011). Physical activity of Canadian adults: Accelerometer results from the 2007 to 2009 Canadian Health Measures Survey. Health Reports, 22, 7–14. 21510585

[gnag173-B15] Costa S. , Oliva P., Cuzzocrea F. (2014). Motivational aspects and personality correlates of physical exercise behavior. *Facta Universitatis, Series: Physical Education and Sport* 83–93.

[gnag173-B16] Courneya K. S. , Hellsten L.-A. M. (1998). Personality correlates of exercise behavior, motives, barriers, and preferences: An application of the five-factor model. Personality and Individual Differences, 24, 625–633. 10.1016/S0191-8869(97)00231-6

[gnag173-B17] Deary I. J. , Weiss A., Batty G. D. (2010). Intelligence and personality as predictors of illness and death: How researchers in differential psychology and chronic disease epidemiology are collaborating to understand and address health inequalities. Psychological Science in the Public Interest: Journal of the American Psychological Society, 11, 53–79. 10.1177/1529100610387081 26168413

[gnag173-B18] Debast I. , van Alphen S. P., Rossi G., Tummers J. H., Bolwerk N., Derksen J. J., Rosowsky E. (2014). Personality traits and personality disorders in late middle and old age: Do they remain stable? A literature review. Clinical Gerontologist, 37, 253–271. 10.1080/07317115.2014.885917

[gnag173-B19] Duberstein P. R. , Sörensen S., Lyness J. M., King D. A., Conwell Y., Seidlitz L., Caine E. D. (2003). Personality is associated with perceived health and functional status in older primary care patients. Psychology and Aging, 18, 25–37. 10.1037/0882-7974.18.1.25 12641310

[gnag173-B20] Emile M. , Chalabaev A., Stephan Y., Corrion K., d’Arripe-Longueville F. (2014). Aging stereotypes and active lifestyle: Personal correlates of stereotype internalization and relationships with level of physical activity among older adults. Psychology of Sport and Exercise, 15, 198–204. 10.1016/j.psychsport.2013.11.002

[gnag173-B21] Erikson E. H. (1994). Identity and the life cycle. WW Norton & Company.

[gnag173-B22] Friedman H. S. , Kern M. L. (2014). Personality, well-being, and health. Annual Review of Psychology, 65, 719–742. 10.1146/annurev-psych-010213-115123 24405364

[gnag173-B23] Ganann R. , Ciliska D., Thomas H. (2010). Expediting systematic reviews: Methods and implications of rapid reviews. Implementation Science: Is, 5, 56. 10.1186/1748-5908-5-56 20642853 PMC2914085

[gnag173-B24] Griffith L. E. , Raina P., Levasseur M., Sohel N., Payette H., Tuokko H., Van Den Heuvel E., Wister A., Gilsing A., Patterson C. (2017). Functional disability and social participation restriction associated with chronic conditions in middle-aged and older adults. Journal of Epidemiology and Community Health, 71, 381–389. 10.1136/jech-2016-207982 27754857

[gnag173-B25] Hansen B. H. , Kolle E., Steene‐Johannessen J., Dalene K. E., Ekelund U., Anderssen S. A. (2019). Monitoring population levels of physical activity and sedentary time in Norway across the lifespan. Scandinavian Journal of Medicine & Science in Sports, 29, 105–112. 10.1111/sms.13314 30276928

[gnag173-B26] Hill K. D. , Hunter S. W., Batchelor F. A., Cavalheri V., Burton E. (2015). Individualized home-based exercise programs for older people to reduce falls and improve physical performance: A systematic review and meta-analysis. Maturitas, 82, 72–84. 10.1016/j.maturitas.2015.04.005 25989701

[gnag173-B27] Hong Q. N. , Fàbregues S., Bartlett G., Boardman F., Cargo M., Dagenais P., Gagnon M.-P., Griffiths F., Nicolau B., O’Cathain A., Rousseau M.-C., Vedel I., Pluye P. (2018). The Mixed Methods Appraisal Tool (MMAT) version 2018 for information professionals and researchers. Education for Information, 34, 285–291. 10.3233/EFI-180221

[gnag173-B28] John O. P. , Srivastava S. (1999). The big five trait taxonomy: History, measurement, and theoretical perspectives. In Pervin L. A., John O. P. (Ed.), Handbook of Personality: Theory and Research. (2nd ed., pp. 102–138). Guilford Press.

[gnag173-B29] Kekäläinen T. , Karvonen J., Törmäkangas T., Pulkkinen L., Kokko K. (2023). Pathways from childhood socioemotional characteristics and cognitive skills to midlife health behaviors. Psychology & Health, 38, 1683–1701. 10.1080/08870446.2022.2041639 35225111

[gnag173-B30] Kekäläinen T. , Laakkonen E. K., Terracciano A., Savikangas T., Hyvärinen M., Tammelin T. H., Rantalainen T., Törmäkangas T., Kujala U. M., Alen M., Kovanen V., Sipilä S., Kokko K. (2020). Accelerometer-measured and self-reported physical activity in relation to extraversion and neuroticism: A cross-sectional analysis of two studies. BMC Geriatrics, 20, 264. 10.1186/s12877-020-01669-7 32727379 PMC7391808

[gnag173-B31] Kekäläinen T. , Luchetti M., Aschwanden D., Sutin A. R., Terracciano A. (2022a). Individual and country-level factors associated with self-reported and accelerometer-based physical activity in old age: A cross-national analysis of European countries. European Journal of Aging, 19, 1529–1542. 10.1007/s10433-022-00737-8 36311335 PMC9589794

[gnag173-B32] Kekäläinen T. , Tammelin T. H., Hagger M. S., Lintunen T., Hyvärinen M., Kujala U. M., Laakkonen E. K., Kokko K. (2022b). Personality, motivational, and social cognition predictors of leisure-time physical activity. Psychology of Sport and Exercise, 60, 102135. 10.1016/j.psychsport.2022.102135

[gnag173-B33] Kekäläinen T. , Terracciano A., Sipilä S., Kokko K. (2020). Personality traits and physical functioning: A cross-sectional multimethod facet-level analysis. European Review of Aging and Physical Activity: Official Journal of the European Group for Research into Elderly and Physical Activity, 17, 20. 10.1186/s11556-020-00251-9 33292163 PMC7685629

[gnag173-B34] Komarraju M. , Karau S. J., Schmeck R. R. (2009). Role of the Big Five personality traits in predicting college students’ academic motivation and achievement. Learning and Individual Differences, 19, 47–52. 10.1016/j.lindif.2008.07.001

[gnag173-B35] Kreuter M. W. , Strecher V. J., Glassman B. (1999). One size does not fit all: The case for tailoring print materials. Annals of Behavioral Medicine: A Publication of the Society of Behavioral Medicine, 21, 276–283. 10.1007/BF02895958 10721433

[gnag173-B36] Laborde S. , Allen M. S., Katschak K., Mattonet K., Lachner N. (2020). Trait personality in sport and exercise psychology: A mapping review and research agenda. International Journal of Sport and Exercise Psychology, 18, 701–716. 10.1080/1612197X.2019.1570536

[gnag173-B37] Lachman M. E. , Teshale S., Agrigoroaei S. (2015). Midlife as a pivotal period in the life course: Balancing growth and decline at the crossroads of youth and old age. International Journal of Behavioral Development, 39, 20–31. 10.1177/0165025414533223 25580043 PMC4286887

[gnag173-B38] Ma Q.-S. , Yao S.-J., Jia H.-R. (2022). The effect of exercise intention on exercise behavior in the post-epidemic era: The moderator role of openness personality and the mediated role of exercise-induced feelings. Frontiers in Psychology, 13, 1050918. 10.3389/fpsyg.2022.1050918 36600711 PMC9807215

[gnag173-B39] Macali I. C. , Smith L., Dale M., Lind E., DeShong H. L., Holmes M. E. (2025). Influences of motivation and personality on physical activity behavior: A systematic review. Journal of Sports Sciences, 43, 623–635. 10.1080/02640414.2025.2468998 39972665

[gnag173-B40] Marks G. R. , Lutgendorf S. K. (1999). Perceived health competence and personality factors differentially predict health behaviors in older adults. Journal of Aging and Health, 11, 221–239. 10.1177/089826439901100205 10558436

[gnag173-B41] McAdams D. P. (1995). What do we know when we know a person? Journal of Personality, 63, 365–396. 10.1111/j.1467-6494.1995.tb00500.x

[gnag173-B42] McCrae R. R. , Costa P. T. Jr. (2008). The five-factor theory of personality. In Handbook of Personality: Theory and Research (3rd ed.). (pp. 159–181). The Guilford Press.

[gnag173-B43] McCrae R. R. , Costa P. T. (1992). Revised NEO Personality Inventory (NEO-PI-R) and NEO Five-Factor Inventory (NEO-FFI) professional manual. *Psychological Assessment Resources, Odessa.*

[gnag173-B44] McEwan D. , Boudreau P., Curran T., Rhodes R. E. (2019). Personality traits of high-risk sport participants: A meta-analysis. Journal of Research in Personality, 79, 83–93. 10.1016/j.jrp.2019.02.006

[gnag173-B45] Michie S. , Van Stralen M. M., West R. (2011). The behavior change wheel: A new method for characterizing and designing behavior change interventions. Implementation Science, 6, 42. 10.1186/1748-5908-6-42 21513547 PMC3096582

[gnag173-B46] Moher D. , Liberati A., Tetzlaff J., Altman D. G.; Group, P. (2009). Preferred reporting items for systematic reviews and meta-analyses: The PRISMA statement. BMJ (Clinical Research ed.), 339, b2535. 10.1136/bmj.b2535 PMC2714657 19622551

[gnag173-B47] Mroczek D. K. , Weston S. J., Willroth E. C. (2020). A lifespan perspective on the interconnections between personality, health, and optimal aging. In P. L. Hill & M. Allemand (Eds.), Personality and healthy aging in adulthood: New directions and techniques (Vol. 26, pp. 191–202). Springer. 10.1007/978-3-030-32053-9_12

[gnag173-B48] Olaru G. , Laukka E. J., Dekhtyar S., Sarwary A., Brehmer Y. (2023). Association between personality traits, leisure activities, and cognitive levels and decline across 12 years in older adults. Psychology and Aging, 38, 277–290. 10.1037/pag0000743 37036694

[gnag173-B49] Petersen G. L. , Mortensen E. L., Rod N. H., Lange T., Flensborg-Madsen T., Hansen Å. M., Lund R. (2018). Occupational social class and personality traits in relation to leisure-time physical activity level: Cross-sectional results from the Copenhagen Aging and Midlife Biobank. Journal of Aging and Health, 30, 1263–1283. 10.1177/0898264317714928 28752788

[gnag173-B50] Rhodes R. E. , Boudreau P. (2017). Physical activity and personality traits. In *the Oxford Research Encyclopedia of Psychology.* 10.1093/acrefore/9780190236557.013.210

[gnag173-B51] Rhodes R. E. , Courneya K. S., Hayduk L. A. (2002). Does personality moderate the theory of planned behavior in the exercise domain? Journal of Sport and Exercise Psychology, 24, 120–132. 10.1123/jsep.24.2.120

[gnag173-B52] Rhodes R. E. , Dickau L. (2013). Moderators of the intention-behavior relationship in the physical activity domain: A systematic review. British Journal of Sports Medicine, 47, 215–225. 10.1136/bjsports-2011-090411 22278998

[gnag173-B53] Rhodes R. E. , Smith N. (2006). Personality correlates of physical activity: A review and meta-analysis. British Journal of Sports Medicine, 40, 958–965. 10.1136/bjsm.2006.028860 17124108 PMC2577457

[gnag173-B54] Roberts B. W. , Walton K. E., Bogg T. (2005). Conscientiousness and health across the life course. Review of General Psychology, 9, 156–168. 10.1037/1089-2680.9.2.156

[gnag173-B55] Robinson A. , Divers R., Moscardini E., Calamia M. (2021). Perfectionism, conscientiousness, and neuroticism: Does age matter? Personality and Individual Differences, 172, 110563. 10.1016/j.paid.2020.110563

[gnag173-B56] Rudnicka E. , Napierała P., Podfigurna A., Męczekalski B., Smolarczyk R., Grymowicz M. (2020). The World Health Organization (WHO) approach to healthy aging. Maturitas, 139, 6–11. 10.1016/j.maturitas.2020.05.018 32747042 PMC7250103

[gnag173-B57] Saul J. E. , Willis C. D., Bitz J., Best A. (2013). A time-responsive tool for informing policymaking: Rapid realist review. Implementation Science, 8, 103. 10.1186/1748-5908-8-103 24007206 PMC3844485

[gnag173-B58] Sjöberg L. , Triolo F., Saadeh M., Dekhtyar S., Calderón-Larrañaga A., Welmer A.-K. (2022). Factors associated with physical activity reduction in Swedish older adults during the first COVID-19 outbreak: A longitudinal population-based study. European Review of Aging and Physical Activity: Official Journal of the European Group for Research into Elderly and Physical Activity, 19, 9. 10.1186/s11556-022-00287-z 35365065 PMC8972725

[gnag173-B59] Stephan Y. (2009). Openness to experience and active older adults’ life satisfaction: A trait and facet-level analysis. Personality and Individual Differences, 47, 637–641. 10.1016/j.paid.2009.05.025

[gnag173-B60] Stephan Y. , Boiché J., Canada B., Terracciano A. (2014). Association of personality with physical, social, and mental activities across the lifespan: Findings from US and French samples. British Journal of Psychology (London, England: 1953), 105, 564–580. 10.1111/bjop.12056 24182200

[gnag173-B61] Stephan Y. , Sutin A. R., Luchetti M., Bosselut G., Terracciano A. (2018). Physical activity and personality development over twenty years: Evidence from three longitudinal samples. Journal of Research in Personality, 73, 173–179. 10.1016/j.jrp.2018.02.005 29651189 PMC5892442

[gnag173-B62] Steptoe A. , Easterlin E., Kirschbaum C. (2017). Conscientiousness, hair cortisol concentration, and health behavior in older men and women. Psychoneuroendocrinology, 86, 122–127. 10.1016/j.psyneuen.2017.09.016 28950115

[gnag173-B63] Stieger M. , Allemand M., Lachman M. E. (2023). Effects of a digital self-control intervention to increase physical activity in middle-aged adults. Journal of Health Psychology, 28, 984–996. 10.1177/13591053231166756 37042306 PMC10466994

[gnag173-B64] Sutin A. R. , Stephan Y., Luchetti M., Artese A., Oshio A., Terracciano A. (2016). The five-factor model of personality and physical inactivity: A meta-analysis of 16 samples. Journal of Research in Personality, 63, 22–28. 10.1016/j.jrp.2016.05.001 29056783 PMC5650243

[gnag173-B65] Thøgersen-Ntoumani C. , Stenling A., Izett E., Quested E. (2022). Personality, risk perceptions, and health behaviors: A two-wave study on reciprocal relations in adults. International Journal of Environmental Research and Public Health, 19, 16168. 10.3390/ijerph192316168 36498240 PMC9740711

[gnag173-B66] Tricco A. C. , Antony J., Zarin W., Strifler L., Ghassemi M., Ivory J., Perrier L., Hutton B., Moher D., Straus S. E. (2015). A scoping review of rapid review methods. BMC Medicine, 13, 224. 10.1186/s12916-015-0465-6 26377409 PMC4574114

[gnag173-B67] Turiano N. A. , Pitzer L., Armour C., Karlamangla A., Ryff C. D., Mroczek D. K. (2012). Personality trait level and change as predictors of health outcomes: Findings from a national study of Americans (MIDUS). The Journals of Gerontology, Series B: Psychological Sciences and Social Sciences, 67, 4–12. 10.1093/geronb/gbr072 21765062 PMC3410684

[gnag173-B68] United Nations, Department of Economic and Social Affairs, Population Division. (2020). *World Population Aging 2019*. https://www.un.org/en/development/desa/population/publications/pdf/aging/WorldPopulationAging2019-Report.pdf

[gnag173-B69] Wang J.-K. , Shao Y. R., Guo L., Mao Z. X. (2024). Personality profiles and exercise behavior across adulthood. Social and Personality Psychology Compass, 18, e12989. 10.1111/spc3.12989

[gnag173-B70] Wilson K. E. , Dishman R. K. (2015). Personality and physical activity: A systematic review and meta-analysis. Personality and Individual Differences, 72, 230–242. 10.1016/j.paid.2014.08.023

[gnag173-B71] World Health Organization (2020). Global guidelines on physical activity and sedentary behavior. W. H. Organization. https://iris.who.int/bitstream/handle/10665/336656/9789240015128-eng.pdf? sequence=1

[gnag173-B72] Yasunaga A. , Yaguchi K. (2014). Personality traits, self-efficacy for exercise, and exercise levels in older Japanese adults. The Japanese Journal of Health Psychology, 27, 1–11. 10.11560/jahp.27.1_1

[gnag173-B73] Zaitsu K. , Nishimura Y., Matsuguma H., Higuchi S. (2015). Association between extraversion and exercise performance among elderly persons receiving a videogame intervention. Games for Health Journal, 4, 375–380. 10.1089/g4h.2014.0119 26287928

